# Supplementary material for: Representation of time interval entrained by periodic stimuli in the visual thalamus of pigeons
Source: eLife. 2017 Dec 29;6:e27995. doi: 10.7554/eLife.27995 (PMC5747522; doi:10.7554/eLife.27995)
Supplement: Transparent reporting form [file elife-27995-transrepform.docx]

***eLife’s* transparent reporting form**

We encourage authors to provide detailed information *within their submission* to facilitate the interpretation and replication of experiments. If you have any questions, please contact us: [editorial@elifesciences.org](mailto:editorial@elifesciences.org).

**Sample-size estimation**

- You should state whether an appropriate sample size was computed when the study was being designed
- You should state the statistical method of sample size computation and any required assumptions
- If no explicit power analysis was used, you should describe how you decided what sample (replicate) size (number) to use

Please outline where this information can be found within the submission (e.g., page numbers or figure legends), or explain why this information doesn’t apply to your submission:

Answers:

1. To balance the sample size and 3R rules, we planned to use 30-40 adult pigeons in the experiment design. Given that the single cell should be luminance sensitive and remained stable recording for 3-4 hours, the number of cells could be recorded from each animal was very limited. We proposed to get enough cells (~100cells) in the avian visual thalamus from these animals under three test conditions (pallium/cortex intact, pallium/cortex pharmacological inactivation and pallium/cortex lesion).
2. In the end, we totally used 38 animals in three experiments, which included 19 animals for the pallium intact, 10 animals for pallium pharmacological inactivation and 9 animals for pallium lesion.
3. We totally recorded 85 cells for further analyses, which completely satisfied the minimum number of samples (n=15) required for two-tailed t-test and one-way ANOVA.

**Replicates**

- You should report how often each experiment was performed
- You should include a definition of biological versus technical replication
- The data obtained should be provided and sufficient information should be provided to indicate the number of independent biological and/or technical replicates
- If you encountered any outliers, you should describe how these were handled
- Criteria for exclusion/inclusion of data should be clearly stated
- High-throughput sequence data should be uploaded before submission, with a private link for reviewers provided (these are available from both GEO and ArrayExpress)

Please outline where this information can be found within the submission (e.g., page numbers or figure legends), or explain why this information doesn’t apply to your submission:

Answers:

1. To analyze the responses of thalamic cells to the periodic stimulus, we isolate a single cell in the brain at first, and then apply the stimulus to this cell and simultaneously record its responses before, during and after stimulus. We measured 190 cells in the same way that were isolated from 19 animals (p.5-6, 32-33). 54 cells encoded the ambient luminance and were used for further analyses. With the same criteria, we also recorded 12 cells from Wulst-inactivation animals and 29 cells from Wulst-lesion animals, respectively.
2. The single-unit recording system is a very developed technology and commonly used in several labs, and the recording system applied in our lab has also been previously described in many papers (p.31-33). The commercial software (spike2, CED) is used to isolate single cell (p.33). Matlab toolbox is used to data analysis (p.34-35, such as FFT analysis, butterworth filter, histogram). In addition, the pigeon used in the experiment is a common animal model in several research area (p.4). The animal care and use follows the standard guidelines of IACUC from CAS (p.31).
3. For each analysis, the corresponding number of independent sample size is given (p. 9-19). Furthermore, we provide the source data of each figure, which can be used to replicate our results (below).
4. In the results and material section, we describe in details the criteria for exclusion/inclusion of data. For example, how to discriminate the recording is multi- or single-unit (p.33-34), whether the recorded cell is luminance sensitive (p.33). In addition, we used the neuronal responses of each cell to photic stimulus to define its cell type (light-activated or light-suppressed, p.5, Figure1) and potential function (replay and non-replay cell, p.6, Figure1).
5. The case of outliers or abnormality in the present experiment occur mainly in the description of cell response pattern. When the periodic stimulus is introduced in the experiment, some cells displayed rhythmic firing patterns which was hidden in the continuously recoded raw data. To avoid the distortion of irregular firing in distinguishing response pattern of each cell, we apply FFT analysis on the response histogram of each cell, and then compare the FFT result with the control estimated by a 1,000 Bootstrap re-sampling of the original response histogram. The detected rhythmic frequency is significant only when FFT amplitude was higher than mean + 4 SD of the control (p.6-8, Figure1). Furthermore, the response histogram is smoothed by zero-phase low-pass Butterworth filter for the further calculation of oscillation duration, and so on (p.6-8, Figure1; p.11-12, Figure3).
6. The avian Wulst in both hemispheres were pharmacologically inactivated by muscimol (2%), which was commonly used in several works and their effective spread in the brain were also carefully measure in previous studies (Figure4). Furthermore, the electrolytic lesions were applied in the Wulst of both hemisphere to exclude the possible incomplete inactivation. Both experiments were carried on anesthetized animals.
7. In the end, we use the computational model to investigate the potential mechanism underlying the unique responses of thalamic cells to periodic stimulus. The model neuron is constructed based on the classic Morris-Lecar model in the textbook. Prof. Izhikevich has ever given deep discussion in his book (Izhikevich, 2007) and source code in matlab. In the manuscript, we give the values of all parameters (figure6, p.26-27).
8. No high-throughput sequence data is used.

**Statistical reporting**

- Statistical analysis methods should be described and justified
- Raw data should be presented in figures whenever informative to do so (typically when N per group is less than 10)
- For each experiment, you should identify the statistical tests used, exact values of N, definitions of center, methods of multiple test correction, and dispersion and precision measures (e.g., mean, median, SD, SEM, confidence intervals; and, for the major substantive results, a measure of effect size (e.g., Pearson's r, Cohen's d)
- Report exact p-values wherever possible alongside the summary statistics and 95% confidence intervals. These should be reported for all key questions and not only when the p-value is less than 0.05.

Please outline where this information can be found within the submission (e.g., page numbers or figure legends), or explain why this information doesn’t apply to your submission:

Answers:

1. All statistical analysis methods should be described and justified in the result section (p. 5-20)
2. N per group in the manuscript is larger than 10. In addition, raw data shown in the figure is provided (p.5-20, below).
3. For each analysis, we give the statistical tested, N values, all exact p-values, precision measures and so on (p.5-20)

(For large datasets, or papers with a very large number of statistical tests, you may upload a single table file with tests, Ns, etc., with reference to page numbers in the manuscript.)

**Additional data files (“source data”)**

- We encourage you to upload relevant additional data files, such as numerical data that are represented as a graph in a figure, or as a summary table
- Where provided, these should be in the most useful format, and they can be uploaded as “Source data” files linked to a main figure or table
- Include model definition files including the full list of parameters used
- Include code used for data analysis (e.g., R, MatLab)
- Avoid stating that data files are “available upon request”

Please indicate the figures or tables for which source data files have been provided:

Answers:

1. We provided all source data for figure1-6.
2. Data is given in excel.
3. The computational model was described in the Materials and methods, and all values of parameters in the model was listed in the figure6’s legend and Materials and methods.
